# Role of Perceived Social Support in COVID-19 Vaccine Uptake Among U.S. Adults

**DOI:** 10.1016/j.focus.2023.100104

**Published:** 2023-04-27

**Authors:** Biplab Kumar Datta, Jennifer E. Jaremski, Benjamin E. Ansa, Lorriane A. Odhiambo, K. M. Monirul Islam, J. Aaron Johnson

**Affiliations:** 1 Institute of Public and Preventive Health, Augusta University, Augusta, Georgia; 2 Department of Population Health Sciences, Medical College of Georgia, Augusta University, Augusta, Georgia

**Keywords:** COVID-19, vaccine uptake, social support, U.S

## Abstract

**Introduction:**

Vaccine uptake concerns in the U.S. were at the forefront of public health discussions during the COVID-19 pandemic. By the end of 2022, approximately 80% of the U.S. population was vaccinated against the virus. This study examined the relationship between perceived social support and COVID-19 vaccine uptake among U.S. adults.

**Methods:**

Using nationally representative cross-sectional data on 21,107 adults from the 2021 National Health Interview Survey, we assessed the COVID-19 vaccination rates across individuals with strong, some, and weak levels of social support. Multivariable logistic regression models were estimated to obtain the odds of being vaccinated in adults with different levels of perceived social support for the full sample and subsamples of age groups.

**Results:**

We found that adults with weak social support were 21.1% less likely to be vaccinated against COVID-19 than adults with perceived strong social support. Apart from the ages 18–24 years group, the lower likelihood of being vaccinated for adults with weak social support was evident in ages 24–49 years (AOR=0.66, 95% CI=0.52, 0.85), ages 50–64 years (AOR=0.67, 95% CI=0.50, 0.90), and ages ≥65 years (AOR=0.56, 95% CI=0.41, 0.75) groups.

**Conclusions:**

These findings are consistent with a broader literature indicating that social support increases the likelihood of healthy behaviors and decreases risky behaviors. Interventions designed to improve the perception of social support, particularly among those at high risk of mortality from COVID-19, may be a promising tactic for increasing COVID-19 vaccine uptake.

## HIGHLIGHTS

• Perceived social support plays an important role in COVID-19 vaccine uptake.
• Lower levels of social support are associated with lower COVID-19 vaccine uptake.
• The association is more evident among older adults.

## Graphical abstract

**Figure:**
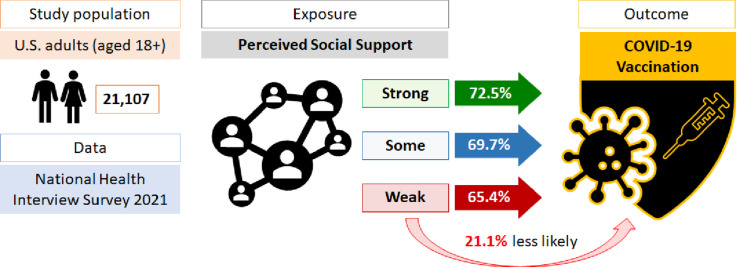

## INTRODUCTION

The coronavirus disease 2019 (COVID-19) pandemic has taken more than 1 million lives in the U.S., while community-level case rates remain high across counties. Currently, the Centers for Disease Control and Prevention reports that COVID-19 infection, hospitalization, and death rates are leveling off nationwide.^1^ During the later months of 2022, both weekly cases and deaths per 100,000 population substantially decreased from respective peaks in January 2022. Weekly death rate declined from 5.23 to 0.76, and weekly case rate declined from 1,696 to 121 from January 2022 to December 2022.^1^ This is because of the protections offered by the available COVID-19 vaccines and boosters.

In response to the outbreak of COVID-19 and its rapid spread across the world, vaccine development jumped to the top of the public health agenda in a race to contain the highly contagious and deadly virus. Emergency Use Authorizations granted by the Food and Drug Administration to Pfizer-BioNTech in December 2020 accelerated the timeline for vaccine distribution after a randomized, controlled, blinded clinical trial with thousands of recipients produced data that showed the safety and effectiveness of the company's first COVID-19 vaccine.^2^ Since then, 79.1% of the U.S. population has received at least 1 dose of the approved vaccines and boosters, including over 1 million children aged <5 years.^1^

Several studies investigated the factors associated with COVID-19 vaccine uptake. A meta-analysis identified sex, educational attainment, influenza vaccination history, and trust in the government as important predictors of intent to get vaccinated for COVID-19.^3^ Studies also reported disparities in COVID-19 vaccination among racial and ethnic minorities.^4^^,^^5^ Furthermore, socioeconomic factors such as education, employment, and income were associated with the disparities in vaccination coverage.^6^ Although vaccine hesitancy has been a major obstacle to COVID-19 vaccine coverage,^7^ a recent study found that vaccine hesitancy did not fully explain the disparities in COVID-19 vaccination coverage in the U.S.^8^ Against this backdrop, we assessed the role of a critical psychosocial factor, social support, on COVID-19 vaccine uptake.

The association between social support and health outcomes has been widely studied in extant literature.^9^ Mortality, mental health, depression and suicidality, tobacco and alcohol use, obesity, and the spread of sexually transmitted diseases are among many health outcomes that have been studied in relation to social support.^10^ Although there is a paucity of existing literature, some published studies provide evidence for the relationship between social support and vaccine uptake. A study of employees of a German company reported a positive association between social support and influenza vaccination.^11^ Similar associations were also reported between social support and influenza, human papillomavirus infection, and shingles vaccine uptake.^12^, ^13^, ^14^ More recently, research with a small sample of respondents in the United Kingdom reported the level of social support being a determinant of the likelihood of vaccination against COVID-19.^15^ Another study reported that social support partially mediated the relationship between COVID-19−related burnout and willingness of booster vaccination in a group of fully vaccinated nurses in Greece.^16^ Influence of social support networks on hesitancy toward COVID-19 vaccination was also found in a qualitative study on older adults in Hong Kong.^17^ Although these studies provide important insights into the association between social support and vaccine uptake, a common limitation of all these studies is that they were conducted on very specific groups (e.g., nurses in a healthcare facility), and the findings thereby were not generalizable.

Therefore, the aim of this study is to assess the differences in COVID-19 vaccination rates among U.S. respondents with varying levels of perceived social support in a nationally representative sample. On the basis of the relationship between social support and health outcomes reported in the extant literature, we hypothesized that COVID-19 vaccination coverage will be lower among respondents who perceive lower levels of social support than among respondents reporting higher levels of social support.

## METHODS

### Study Sample

This cross-sectional study used secondary data from the 2021 wave of the National Health Interview Survey (NHIS). The NHIS is a nationally representative household survey that collects information on various health issues of the U.S. civilian non-institutionalized population in 2 groups: Sample Adults and Sample Children. The NHIS data are widely used by the U.S. government agencies to monitor trends in illness and disability and by the public health research community for policy analysis, program evaluation, and research on a wide range of public health issues in the U.S.^18^ In 2021, interviews were conducted by combinations of telephone and personal visits using computer-assisted personal interviewing data collection method. The Sample Adult response rate was 50.9%. Detailed survey methods of the NHIS, including sample design and interviewing procedures, are available in the 2021 NHIS Survey Description from the National Center for Health Statistics.^18^ From the Sample Adult interviews of the NHIS, we obtained data on 21,107 adult individuals for whom COVID-19 vaccination and social support information were available. Because we used publicly available anonymized secondary data, ethics committee approval was not required.

### Measures

The NHIS asked respondents whether they had a COVID-19 vaccination (at least 1 dose). Respondents who answered yes to this question were considered COVID-19 vaccinated. Our dependent variable, thus, is a binary variable indicating whether a respondent was COVID-19 vaccinated or not.

The NHIS asked respondents, *How often do you get the social and emotional support you need? Would you say always, usually, sometimes, rarely, or never?*^18^ The responses *always* or *usually* were regarded as strong social support, the response *sometimes* was regarded as some social support, and the responses *rarely* or *never* were regarded as weak social support. Our key explanatory variable thus is a polychotomous variable indicating an overall strong, medium, or weak level of perceived social support. Of note, respondents in the NHIS were not asked about COVID-19−specific support, but social and emotional support in general.

### Statistical Analysis

We first assessed whether the percentage of respondents vaccinated against COVID-19 varied by level of perceived social support by performing adjusted Wald tests. Next, binomial logistic regressions were estimated to obtain the odds in favor of being vaccinated against COVID-19. We reported ORs for some and weak levels of perceived social support, with strong social support serving as the reference category.

Next, we estimated multivariable specifications, where we controlled for various demographic and socioeconomic factors. The demographic characteristics included age (continuous variable), sex (male and female), and race and ethnicity (non-Hispanic White, non-Hispanic Black, non-Hispanic Asian, Hispanic, and non-Hispanic other). The socioeconomic characteristics include marital status (married, never married, living with a partner, and widowed/divorced/separated), educational attainment (college graduate, some college, high school graduate, and less than high school diploma), household income (≥400% of the Federal Poverty Line [FPL], 200 to <400% of the FPL, 100 to <200% of the FPL, and <100% of the FPL), insurance coverage (no and yes), and employment status (not employed and employed). In addition, to account for vaccine availability, we controlled for urban/rural residence captured by urban−rural classification of counties (large central metropolis, large fringe metropolis, medium and small metropolis, and nonmetropolitan), U.S. Census Bureau regions (Northeast, Midwest, South, and West), and interview month fixed effects. Of note, we did not intend to analyze how these correlates were associated with receipt of COVID-19 vaccination. Rather, we were interested in assessing whether the relationship between the levels of social support and COVID-19 vaccination persisted after accounting for these potential confounding variables.

We estimated the models for the full sample and for subsamples of ages 18–24 years, ages 25–49 years, ages 50–64 years, and ages ≥65 years. All estimates were obtained using complex survey weights associated with the stratified cluster sampling design of the NHIS.^18^ Analyses were conducted in Stata 17.0 software.

## RESULTS

Of the 21,107 study respondents, more than 80% reported strong social support. Whereas 11% reported having some social support, and about 7% indicated having weak social support. Table 1 shows the descriptive statistics of the study population by level of perceived social support. Respondents who were married, with some college experience or a college degree, and with household income ≥400% of FPL had a lower proportion reporting weak social support. Non-Hispanic White respondents had a lower proportion of weak social support than other race and ethnicity categories. In contrast, respondents who reported being unemployed, being uninsured, living in large central metropolitan areas, and being from the West had a higher proportion of weak social support.

**Table 1:** Descriptive Statistics

Characteristic	Frequency				Share (%)			
	All	Strong	Some	Weak	All	Strong	Some	Weak
Sex								
Male^a^	9,570	7,713	1,056	801	48.25	48.10	47.00	52.06
Female	11,536	9,471	1,302	763	51.75	51.90	53.00	47.94
Age, years								
18–24	1,317	1,064	165	88	11.52	11.55	12.49	9.66
25–49	7,915	6,442	918	555	41.70	41.74	42.22	40.38
50–64	5,402	4,293	654	455	24.44	23.91	26.18	28.10
≥65	6,473	5,386	621	466	22.34	22.80	19.11	21.86
Race and ethnicity								
White^a^	14,125	11,881	1,419	825	64.89	67.07	58.35	48.42
Black	2,211	1,659	341	211	11.82	10.98	15.58	16.18
Asian	1,285	953	178	154	6.03	5.35	8.00	11.26
Hispanic	2,823	2,191	337	295	16.69	16.08	17.44	23.07
Other	113	84	15	14	0.57	0.52	0.63	1.07
Marital status								
Married^a^	9,911	8,591	846	474	52.09	54.55	42.17	37.72
Never married	4,428	3,340	642	446	23.71	22.48	28.8	30.68
Living with partner	1,354	1,141	133	80	8.53	8.56	8.94	7.61
Widowed/divorced/separated	5,322	4,044	725	553	15.67	14.41	20.10	23.99
Urban/rural area								
Large central metropolitan^a^	6,355	5,064	765	526	31.64	31.07	32.34	37.52
Large fringe metropolitan	5,030	4,205	508	317	24.04	24.66	22.36	19.13
Medium and small metropolitan	6,699	5,454	737	508	30.89	30.8	31.47	31.07
Nonmetropolitan	3,023	2,462	348	213	13.43	13.48	13.83	12.28
Region								
Northeast^a^	3,411	2,770	390	251	17.24	17.23	17.56	16.85
Midwest	4,520	3,772	478	270	21.00	21.5	20.37	15.91
South	7,666	6,261	844	561	38.01	38.07	37.69	37.76
West	5,510	4,382	646	482	23.74	23.19	24.37	29.49
Education								
College graduate^a^	8,254	7,139	738	377	36.1	38.21	28.39	22.39
Some college	5,772	4,642	669	461	26.26	26.12	27.71	25.72
High school graduate	5,177	4,060	655	462	28.24	27.32	31.18	34.85
Less than high school diploma	1,801	1,271	280	250	9.39	8.34	12.72	17.04
Household income								
≥400% of FPL^a^	9,397	8,210	788	399	43.93	46.88	32.90	25.30
200 to <400% of FPL	6,029	4,872	703	454	29.08	28.61	32.28	31.31
100 to <200% of FPL	3,643	2,733	514	396	17.35	16.26	21.59	23.94
<100% of FPL	2,038	1,370	353	315	9.64	8.24	14.23	19.45
Insurance coverage								
No^a^	1,383	968	237	178	8.33	7.31	12.4	14.34
Yes	19,705	16,201	2,119	1,385	91.67	92.69	87.6	85.66
Employment status								
Not employed^a^	8,864	7,048	1,041	775	37.56	36.61	40.23	44.97
Employed	12,163	10,076	1,307	780	62.44	63.39	59.77	55.03

*Note:* N=21,107. Some categories may not add up to 21,107 because of missing values. Shares were obtained using complex survey weights. Shares add to 100 across rows for respective characteristics.

a Reference group. FPL, Federal Poverty Line.

**Figure 1:**
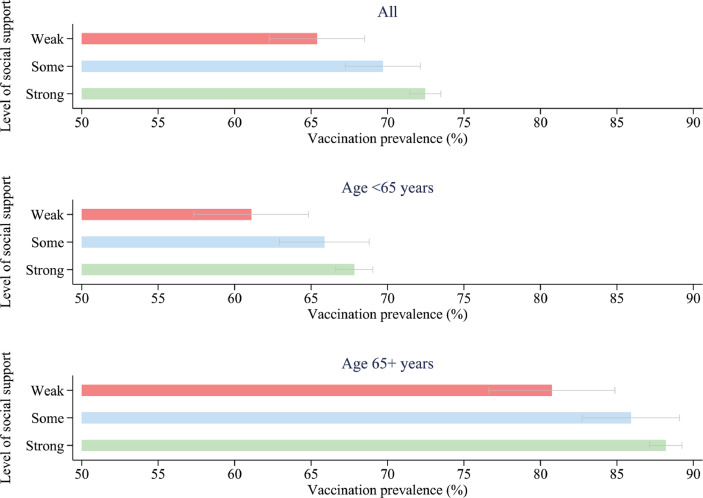
COVID-19 vaccination prevalence by the level of perceived social support.

Around 71.7% of the respondents in our sample were vaccinated against COVID-19. Among those who were vaccinated, 87.5% received more than 1 dose of COVID-19 vaccine. Figure 1 presents the COVID-19 vaccination prevalence by level of perceived social support. Although the vaccination rate was 72.5% among respondents with strong social support, it was 2.8 (95% CI=0.3, 5.2) and 7.1 (95% CI=3.9, 10.2) percentage points lower among those with some and weak social support, respectively. Overall vaccination rates were higher among respondents aged ≥65 years (87.5%) than among respondents aged <65 years (67.1%). In both populations, vaccination rates were significantly higher among respondents with strong social support than among their counterparts who reported weak social support. Vaccination rates for respondents aged <65 years and respondents aged ≥65 years with strong social support were respectively 6.7 (95% CI=2.9, 10.6) and 7.4 (95% CI=3.2, 11.7) percentage points higher than those for respondents with weak social support. However, the differences in vaccination rates between respondents in both age categories with strong and some levels of social support were not statistically significant.

Table 2 reports the unadjusted and adjusted ORs in favor of being vaccinated against COVID-19. Respondents with weak social support were 28.2% less likely to receive a COVID-19 vaccine than respondents with strong social support. When sociodemographic and socioeconomic correlates were accounted for, respondents reporting weak social support were 21.1% less likely to be vaccinated against COVID-19. All demographic and socioeconomic covariates included in the model also showed a significant relationship with vaccination against COVID-19.

**Table 2:** Crude and Adjusted ORs in Favor of Being Vaccinated

Variables	Unadjusted	Adjusted for sociodemographic correlates	Adjusted for sociodemographic and socioeconomic correlates
Social support			
Strong	Ref	Ref	Ref
Some	0.874* (0.778, 0.982)	0.867* (0.763, 0.984)	1.010 (0.883, 1.156)
Weak	0.718*** (0.623, 0.827)	0.629*** (0.542, 0.731)	0.789** (0.678, 0.918)
Sex			
Male	**Ref**	**Ref**	**Ref**
Female	1.097* (1.016, 1.184)	1.130** (1.040, 1.228)	1.118* (1.024, 1.221)
Age	1.032*** (1.030, 1.035)	1.039*** (1.036, 1.042)	1.041*** (1.038, 1.044)
Race			
White	Ref	Ref	Ref
Black	0.702*** (0.616, 0.800)	0.797** (0.686, 0.925)	1.048 (0.902, 1.218)
Asian	3.220*** (2.582, 4.017)	3.057*** (2.399, 3.896)	3.065*** (2.365, 3.972)
Hispanic	0.737*** (0.657, 0.827)	0.833** (0.724, 0.957)	1.481*** (1.267, 1.731)
Other	0.604* (0.381, 0.956)	0.559* (0.348, 0.900)	0.871 (0.554, 1.369)
Marital status			
Married	Ref	Ref	Ref
Never married	0.516*** (0.468, 0.570)	1.006 (.899, 1.126)	1.326*** (1.168, 1.504)
Living with partner	0.383*** (0.337, 0.436)	0.632*** (0.548, 0.728)	0.786** (0.673, 0.917)
Widowed/divorced/separated	0.884* (0.800, 0.977)	0.644*** (0.575, 0.722)	0.806*** (0.717, 0.907)
Area			
Large central metropolitan	Ref	Ref	Ref
Large fringe metropolitan	0.975 (0.857, 1.109)	0.833* (0.719, 0.966)	0.829** (0.720, 0.955)
Medium and small metropolitan	0.702*** (0.621, 0.795)	0.630*** (0.546, 0.725)	0.763*** (0.662, 0.879)
Nonmetropolitan	0.512*** (0.445, 0.590)	0.391*** (0.334, 0.457)	0.554*** (0.474, 0.647)
Region			
Northeast	Ref	Ref	Ref
Midwest	0.667*** (0.574, 0.775)	0.777** (0.660, 0.916)	0.830* (0.707, 0.973)
South	0.547*** (0.472, 0.633)	0.615*** (0.522, 0.725)	0.650*** (0.557, 0.760)
West	0.811* (0.692, 0.951)	0.924 (0.773, 1.105)	0.909 (0.761, 1.086)
Education			
College graduate	Ref		Ref
Some college	0.366*** (0.332, 0.404)		0.452*** (0.402, 0.509)
High school graduate	0.251*** (0.227, 0.277)		0.323*** (0.286, 0.364)
Less than high school diploma	0.250*** (0.217, 0.288)		0.292*** (0.246, 0.346)
Household income			
≥400% of FPL	Ref		Ref
200 to <400% of FPL	0.522*** (0.476, 0.573)		0.677*** (0.607, 0.755)
100 to <200% of FPL	0.350*** (0.314, 0.391)		0.512*** (0.446, 0.588)
<100% of FPL	0.273*** (0.238, 0.314)		0.450*** (0.375, 0.539)
Insurance coverage			
No	Ref		Ref
Yes	3.008*** (2.615, 3.460)		1.570*** (1.344, 1.834)
Employment status			
Not employed	Ref		Ref
Employed	0.820*** (0.756, 0.890)		0.929 (0.837, 1.030)
Observations	21,107	20,469	20,330

*Note:* Boldface indicates statistical significance (***p<0.001, **p<0.01, and *p<0.05).

Estimates were obtained using complex survey weights. The 95% CIs are in parentheses. The multivariable models account for interview months fixed effects.

FPL, Federal Poverty Line.

The relationship between the levels of perceived social support and COVID-19 vaccination uptake was evident in age-related subgroups (Table 3). Respondents aged <65 years and those aged ≥65 years with weak social support were 24.5% and 43.8% less likely to be vaccinated, respectively. After accounting for sociodemographic and socioeconomic correlates, respondents in both age categories with weak social support were 19.1% and 29.9% less likely, respectively, to be vaccinated than their counterparts with strong social support. Within the group of respondents aged <65 years, the lower odds of COVID-19 vaccination for the weak level of social support were not statistically significant. When sociodemographic correlates were accounted for, respondents aged 25–49 and 50–64 years with weak levels of perceived social support were found to be 33.7% and 32.9%, respectively, less likely to receive COVID-19 vaccine. However, the lower odds associated with weak social support in these 2 age groups were not statistically significant when socioeconomic attributes were included in the model.

**Table 3:** Crude and Adjusted ORs in Favor of Being Vaccinated by Age Group

Variables	Age, years 18–24	Age, years 25–49	Age, years 50–64	Age, years <65	Age, years ≥65
Unadjusted					
Social support					
Strong	Ref	Ref	Ref	Ref	Ref
Some	0.996 (0.678, 1.462)	0.856 (0.722, 1.014)	0.965 (0.769, 1.210)	0.916 (0.802, 1.045)	0.816 (0.614, 1.086)
Weak	0.795 (0.485, 1.304)	0.668*** (0.534, 0.836)	0.747* (0.573, 0.975)	0.745*** (0.633, 0.877)	0.562*** (0.424, 0.743)
Observations	1,317	7,915	5,402	14,634	6,473
Adjusted for sociodemographic correlates					
Social support					
Strong	Ref	Ref	Ref	Ref	Ref
Some	0.922 (0.621, 1.368)	0.801* (0.664, 0.967)	0.999 (0.780, 1.278)	0.875 (0.759, 1.008)	0.829 (0.614, 1.119)
Weak	0.671 (0.393, 1.145)	0.663*** (0.520, 0.846)	0.671** (0.498, 0.904)	0.651*** (0.548, 0.774)	0.557*** (0.413, 0.750)
Observations	1,265	7,612	5,261	14,138	6,331
Adjusted for sociodemographic and socioeconomic correlates					
Social support					
Strong	Ref	Ref	Ref	Ref	Ref
Some	1.018 (0.653, 1.587)	0.950 (0.775, 1.163)	1.188 (0.914, 1.544)	1.016 (0.873, 1.182)	0.980 (0.726, 1.325)
Weak	0.809 (0.468, 1.401)	0.881 (0.691, 1.125)	0.868 (0.640, 1.178)	0.819* (0.689, 0.974)	0.701* (0.517, 0.952)
Observations	1,254	7,567	5,217	14,038	6,292

*Note:* Boldface indicates statistical significance (***p<0.001, **p<0.01, and *p<0.05).

Estimates were obtained using complex survey weights. The 95% CIs are in parentheses. Sociodemographic correlates included sex, age, race and ethnicity, marital status, metropolitan area, and region. Socioeconomic correlates included educational attainment, household income, insurance coverage, and employment status. All multivariable specifications account for interview months fixed effects.

## DISCUSSION

Disparities in COVID-19 vaccine uptake in the U.S. have been well documented in extant literature.^19^^,^^20^ However, our knowledge and understanding of the underlying causes of the disparities are continuously evolving. We contribute to this body of literature by assessing the role of perceived social support on the likelihood of being vaccinated against COVID-19. In a nationally representative sample, we found that respondents with lower levels of perceived social support were less likely to be vaccinated against COVID-19 than respondents with higher levels of social support.

During the months between the first Emergency Use Authorizations vaccine and the declaration of COVID-19 as a pandemic by the WHO on March 11, 2020, the public was urged to help mitigate the virus spread through preventive behaviors, such as the use of face coverings, social distancing, lockdowns, frequent handwashing, surface cleaning, and self-quarantining when sick.^21^ After vaccines were made available to the public in early 2021, being vaccinated against the coronavirus was considered an effective preventive measure and a cornerstone of the pandemic response.^22^^,^^23^ Although COVID-19 mortality and morbidity varied by various factors, including pre-existing comorbidities, diet and nutrition, atopic conditions, and demographic characteristics, vaccination was found to be an important protective factor against infection and disease progression.^24^ Therefore, understanding the factors associated with the acceptance and uptake of the COVID-19 vaccine has great public health relevance.

Social influence or social comparison, social control, belonging and companionship, self-esteem, and sense of control among others could be potential pathways through which social support may impact COVID-19 vaccination.^25^ In a systematic review of 47 peer-reviewed articles, psychological and societal aspects such as trust, social influence, fear and anxiety, along with safety, side effects, and effectiveness of the vaccine, were found to be potential factors influencing COVID-19 vaccine acceptance and hesitancy.^26^ Influence of political ideology and affiliation and receiving information from culturally relevant sources also came up as important determinants of decision making in another qualitative study.^27^ These psychosocial factors influencing vaccine uptake can be impacted by different levels of perceived social support. The relationship between vaccine uptake and these factors can be moderated by social support as well. Future research may explore these issues for a nuanced understanding of the role of social support on COVID-19 vaccine uptake.

When we controlled for socioeconomic correlates, such as educational attainment and household income, the association between perceived social support and COVID-19 vaccine uptake became relatively smaller. This may be because perceived social support could be commensurate with socioeconomic conditions.^28^ Except for the age group of 18–24 years, a weak level of social support was found associated with a lower likelihood of vaccine uptake across all other age categories. Among U.S. adults, vaccine uptake was the lowest in the age group 18–24 years, and the percentage of people receiving COVID-19 vaccine gradually increased in older age groups (i.e., ages 25–49, 50–64, and ≥65 years).^1^ The higher vaccine hesitancy in younger age groups and the greater willingness to accept vaccine in older age groups were observed in other countries as well.^29^ Our results suggest that perceived social support played an important role in vaccine uptake among older adults, who tend to have a relatively greater vaccine acceptance. Further research is warranted to explore issues impacting vaccine uptake in younger adults.

Our results are comparable with those of a recent study that suggests that disparities in vaccine uptake in U.S. counties were attributable to differences in social capital across the counties.^30^ On the individual level, social capital in a Japanese population, in the form of individual-level civic participation, social cohesion, and reciprocity, was found associated with the increased odds of getting a COVID-19 vaccine and intention of getting boosted.^31^ Another study found that Italian adults aged 18–40 years who perceived themselves as having less social support from their friends and family were more likely to refuse the COVID-19 vaccine or be hesitant toward it.^32^ Studies also reported a positive role of governmental support and incentives on COVID-19 vaccine uptake.^33^^,^^34^

The critical influence of social support during the COVID-19 vaccination process emerged as a subtheme in a qualitative study that held 8 focus groups with 72 members of the Black and Latinx communities.^35^ Despite this awareness, amidst other factors, the lower levels of perceived social support among Hispanics and non-Hispanic Blacks contributed to hesitancy in this population. These studies that investigated the role of social support during the pandemic mainly focused on the type of social support (i.e., friends, family, community, and social media) but not much on the level of social support. One can be connected to a network of people but still perceive having low levels of informational, functional, or emotional social support. As such, our findings provide important insights for improving vaccine uptake.

### Limitations

However, our study is subject to some limitations. First, there was no time reference point (e.g., in the last 12 months, etc.) for the question asked about social and emotional support among respondents. Second, owing to the cross-sectional nature of the data, we were not able to infer any causal association between social support and COVID-19 vaccine uptake. Third, in the absence of state identifiers, we could not account for state-level variations. Despite these limitations, our analysis is among the few if not the first to report the relationship between levels of perceived social support and COVID-19 vaccine uptake in a nationally representative U.S. sample.

## CONCLUSIONS

Vaccination is regarded as one of the most effective tools to reduce morbidity and mortality from the COVID-19 virus. Yet, vaccine uptake in the U.S. population remained a critical public health challenge. Our results motivate further research to explore the causal link between perceived social support and vaccine uptake to inform policies for enhanced vaccine uptake through engendering social support.

## CRediT authorship contribution statement

**Biplab Kumar Datta:** Conceptualization, Methodology, Software, Validation, Formal analysis, Writing – original draft. **Jennifer E. Jaremski:** Conceptualization, Writing – original draft. **Benjamin E. Ansa:** Validation, Writing – review & editing. **Lorriane A. Odhiambo:** Writing – review & editing. **K. M. Monirul Islam:** Conceptualization, Writing – review & editing. **J. Aaron Johnson:** Writing – review & editing, Supervision.
